# Protective effect of hydrogen sulphide against myocardial hypertrophy in mice

**DOI:** 10.18632/oncotarget.15765

**Published:** 2017-02-28

**Authors:** Mingjing Shao, Chuanjun Zhuo, Ronghuan Jiang, Guangdong Chen, Jianmin Shan, Jing Ping, Hongjun Tian, Lina Wang, Chongguang Lin, Lirong Hu

**Affiliations:** ^1^ National Integrated Traditional and Western Medicine Center for Cardiovascular Disease, China-Japan Friendship Hospital, Beijing, China; ^2^ Department of Psychological Medicine, Wenzhou Seventh People's Hospital, Wenzhou, China; ^3^ Department of Psychological Medicine, Tianjin Anding Hospital, Tianjin, China; ^4^ Department of Psychological Medicine, Tianjin Anning Hospital, Tianjin, China; ^5^ Department of Psychological Medicine, Chinese People's Liberation Army General Hospital, Chinese People's Liberation Army Medical School, Beijing, China

**Keywords:** hydrogen sulfide, cardiac hypertrophy, oxidative stress, Nrf2, Pathology Section

## Abstract

Cardiac hypertrophy is a critical component of phenotype in the failing heart. Recently, increasing evidence has demonstrated that oxidative stress plays an important role in the pathogenesis of myocardial hypertrophy. In the present study, we generated a mouse model of transverse aortic constriction (TAC) to investigate whether hydrogen sulfide (H_2_S) has protective effects against cardiac hypertrophy. Left ventricular structure was analyzed by two-dimensional echocardiography. Oxidative stress was evaluated by measuring malondialdehyde, superoxide dismutase, glutathione peroxidase and reactive oxygen specie in the myocardium. Angiotensin II (Ang-II) was used to induce cardiomyocyte hypertrophy. Neonatal rat cardiomyocytes pretreated with H_2_S donor sodium hydrosulfide prior to Ang-II exposure were used to determine the involvement of Nrf2 and PI3K/Akt pathway in the antioxidant effects of H_2_S. Our findings showed that H_2_S could protect against cardiac hypertrophy by attenuating oxidative stress. The antioxidant roles of H_2_S in myocardial hypertrophy probably depend on the activation of PI3K/Akt signaling, which consequently increases Nrf2 activity and HO-1 and GCLM expression. In summary, H_2_S may exert antioxidant effect on cardiac hypertrophy via PI3K/Akt-dependent activation of Nrf2 pathway.

## INTRODUCTION

Cardiac hypertrophy, which is characterized by myocardial fibrosis, capillary rarefaction, inflammatory reaction, and cellular dysfunction, consequently results in maladaptive ventricular remodeling and heart failure. The mechanical stress and neurohumoral stimulation are primary triggering events of myocardial hypertrophy. These events are associated with various cellular responses including gene transcription, protein translation, sarcomere assembly and cell metabolism, thereby leading to the progression of cardiac hypertrophy [1, 2]. Under normal conditions, ventricular function is initially maintained by cardiac hypertrophy induced by pressure overload, and this is known as the adaptive phase. However, sustained pressure overload can promote the transition from the adaptive to maladaptive phase, which is characterized by left ventricular enlargement and dysfunction [3].

In recent years, hydrogen sulphide (H_2_S) has been recognized as a new gaseous signaling molecule aside from nitric oxide and carbon monoxide. It has multiple physiological and pharmacological properties such as cardioprotection, vasorelaxation, antioxidant and anti-inflammatory effects [4]. In the cardiovascular system, H_2_S is produced in the myocardium and blood vessels from L-cysteine by cystathionine γ-lyase (CSE). Recently, there is growing evidence that H_2_S plays important roles in the pathogenesis of various cardiovascular diseases [5, 6]. H_2_S has been found to attenuate myocardial ischemia-reperfusion injury by preservation of mitochondrial function and to reduce the mortality of heart failure induced by myocardial ischemia [7, 8]. In addition, H_2_S has been shown to improve myocardial fibrosis by inhibiting oxidative stress, blocking TGF-β1/Smad2 signaling pathway, and reducing α-SMA expression in cardiac fibroblasts [9]. In the present study, we generated a mouse model of transverse aortic constriction (TAC) to investigate whether H_2_S has protective effect against cardiac hypertrophy.

## RESULTS

**Figure 1:**
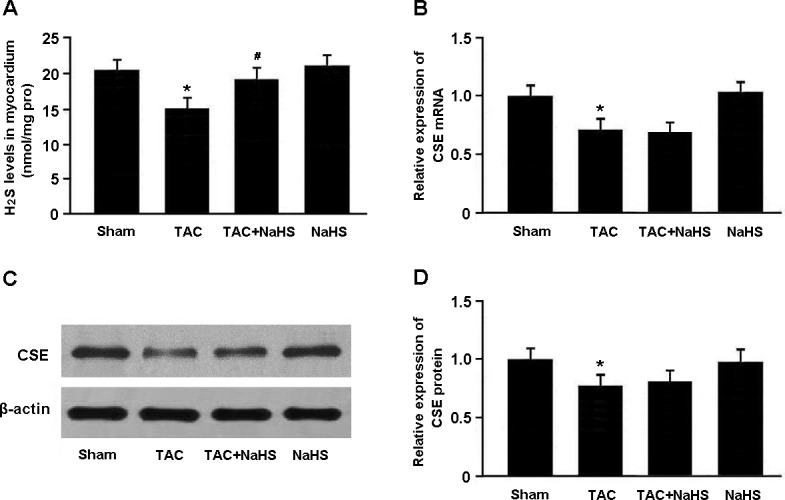
**A**. The H_2_S levels in myocardial tissue were measured by the methylene blue method. **B**.-**D**. The mRNA and protein expression of cystathionine γ-lyase (CSE) was determined by real-time PCR and Western blotting. * *P* < 0.05, vs. Sham; ^#^ *P* < 0.05, vs. TAC (*n* = 5).

H_2_S levels in the myocardium were markedly lower in the TAC group than in the Sham group, while in the TAC+NaHS group, H_2_S levels were significantly elevated compared with in the TAC group (Figure 1A). Moreover, the mRNA and protein expression of CSE was remarkably downregulated in the TAC group compared with in the Sham group (Figure 1B-1D).

**Figure 2:**
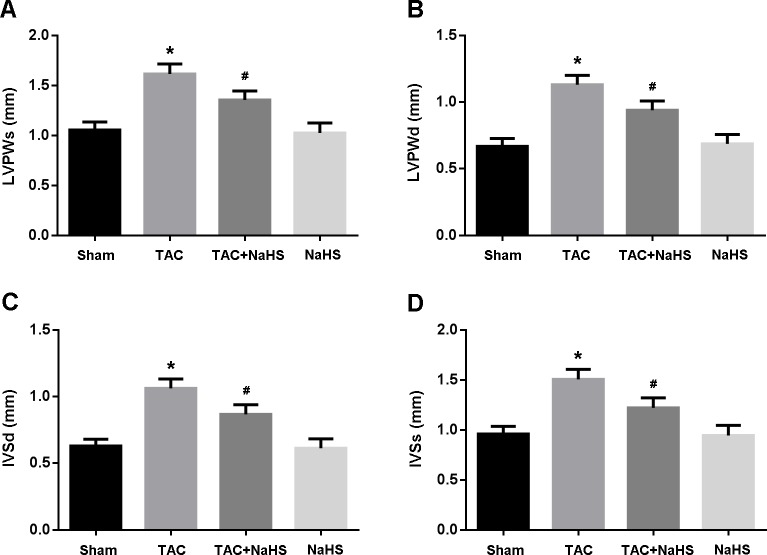
Echocardiographic evaluation of cardiac hypertrophy **A**. Left ventricular posterior wall thickness at end-systole (LVPWs); **B**. Left ventricular posterior wall thickness at end-diastole (LVPWd); **C**. Interventricular septum thickness at end-diastole (IVSd); **D**. Interventricular septum thickness at end-systole (IVSs). * *P* < 0.05, vs. Sham; ^#^ *P* < 0.05, vs. TAC (*n* = 5).

The echocardiography was performed to evaluate cardiac hypertrophy. LVPWd, LVPWs, IVSd and IVSs were significantly increased in the TAC group compared with in the Sham group, while in the TAC+NaHS group, these parameters were remarkably decreased compared with in the TAC group (Figure 2A-2D).

**Figure 3:**
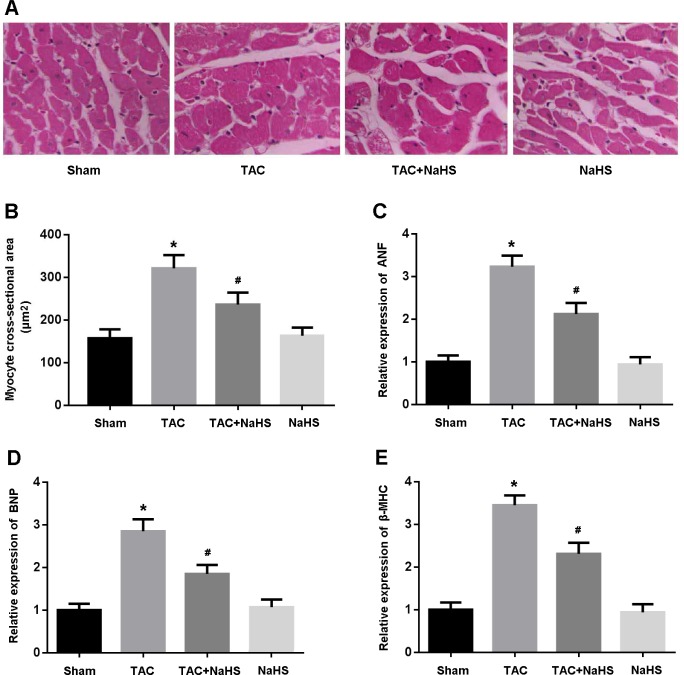
**A**. Representative images of left ventricular tissue sections stained with hematoxylin-eosin; **B**. Quantitative analysis of myocyte cross-sectional area; **C**.-**E**. Relative expression of ANF, BNP and β-MHC detected by real-time PCR. * *P* < 0.05, vs. Sham; ^#^ *P* < 0.05, vs. TAC (*n* = 5).

Left ventricular tissue was stained with HE and CSA was found to be significantly increased in the TAC mice and reduced after treatment with NaHS (Figure 3A, 3B). In addition, the expressions of ANF, BNP and β-MHC, indicators of myocardial hypertrophy, were remarkably increased in the TAC group, while their expressions were markedly decreased following treatment with NaHS (Figure 3C-3E).

**Figure 4:**
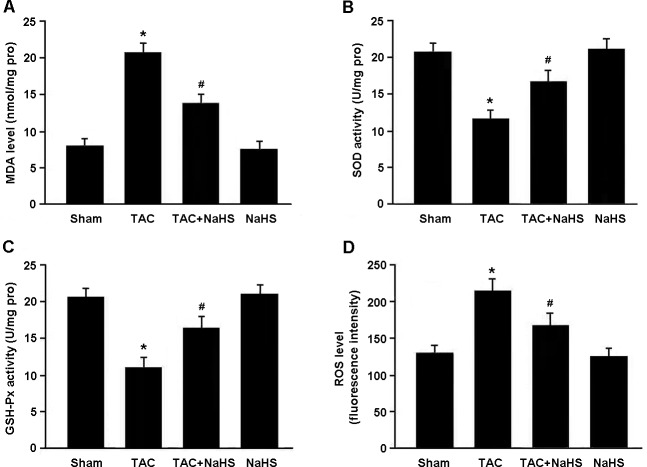
Assessment of oxidative stress in myocardial tissue **A**. Malondialdehyde (MDA) level; **B**. Superoxide dismutase (SOD) activity; **C**. Glutathione peroxidase (GSH-Px) activity; **D**. Reactive oxygen species (ROS) level. * *P* < 0.05, vs. Sham; ^#^ *P* < 0.05, vs. TAC (*n* = 5).

Oxidative stress was evaluated by detecting MDA, SOD, GSH-Px and ROS in the myocardium (Figure 4A-4D). There were significant increase in MDA levels and decrease in SOD and GSH-Px activities in TAC mice, while NaHS treatment was found to reduce MDA levels and enhance SOD and GSH-Px activities. In addition, the ROS generation in myocardial tissue was remarkably elevated in the TAC group and reduced in the TAC+NaHS group.

**Figure 5:**
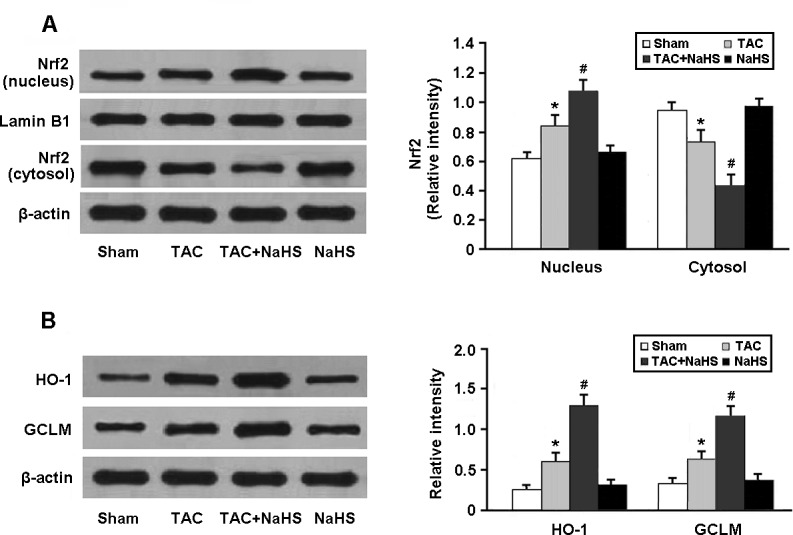
Western blotting analysis of Nrf2 in the nucleus and cytosol (**A**) and its downstream targets HO-1 and GCLM (**B**). * *P* < 0.05, vs. Sham; ^#^ *P* < 0.05, vs. TAC (*n* = 5).

As shown in Figure 5A, the Nrf2 protein accumulated in the myocardial cell nucleus and Nrf2-ARE binding activity was increased in the myocardium of TAC mice after treatment with NaHS. Moreover, the protein expressions of HO-1 and GCLM, downstream targets of Nrf2, were significantly upregulated in the TAC+NaHS group compared with in the TAC group (Figure 5B).

**Figure 6:**
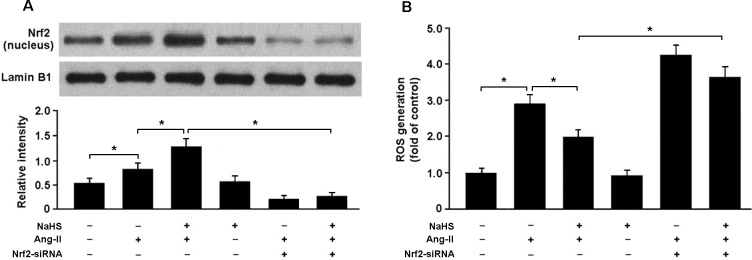
**A**. Western blotting analysis of Nrf2 nuclear protein in cardiomyocytes. **B**. Nrf2 siRNA-transfected cells pretreated with NaHS (100 μM) for 30 min prior to exposure to Ang-II (150 nM) for 24 h exhibited increased ROS production. * *P* < 0.05 (*n*= 5 independent experiments).

To determine whether H_2_S attenuates Ang-II-induced oxidative stress in the Nrf2-dependent manner, we transfected cardiomyocytes with Nrf2 siRNA and then subjected them to Ang-II. Our results indicated that Nrf2 siRNA-transfected cells that had been exposed to Ang-II and NaHS showed decreased nuclear expression of Nrf2 and elevated ROS generation compared with cells that had not been transfected with Nrf2 siRNA (Figure 6A, 6B).

**Figure 7:**
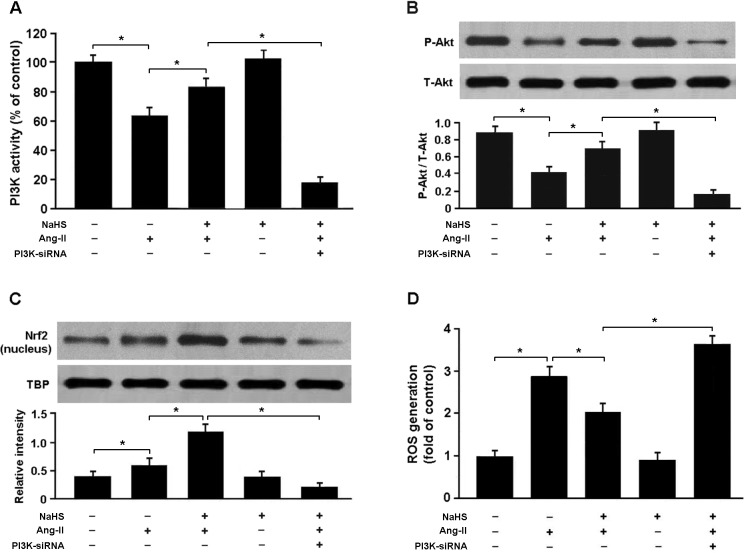
**A**. PI3K activity in cardiomyocytes was detected by enzyme-linked immunosorbent assay. **B**. Western blotting analysis of Akt phosphorylation in cardiomyocytes. **C**. The nuclear protein of Nrf2 were significantly upregulated in cells treated with Ang-II and NaHS and downregulated in PI3K siRNA-transfected cells exposed to Ang-II and NaHS. **D**. PI3K siRNA-transfected cells pretreated with NaHS (100 μM) for 30 min prior to exposure to Ang-II (150 nM) for 24 h exhibited increased ROS production. * *P* < 0.05 (*n* = 5 independent experiments).

To confirm whether H_2_S regulates Nrf2 via PI3K/Akt-dependent pathway, we transfected cardiomyocytes with PI3K siRNA and then subjected them to Ang-II. Our results showed that PI3K siRNA-transfected cells exposed to Ang-II and NaHS exhibited reduced expression of phosphorylated Akt and nuclear Nrf2 and increased production of ROS compared with cells not transfected with PI3K siRNA (Figure 7A-7D).

## DISCUSSION

In this study, we established a mouse model of cardiac hypertrophy using TAC method and found that NaHS treatment could significantly attenuate myocardial hypertrophy and regulate the expression of hypertrophy-associated genes in TAC mice. In addition, we also found that endogenous generation of H_2_S and CSE expression were remarkably decreased in hypertrophic mice, while exogenous supplement of NaHS increased H_2_S levels in the myocardial tissue.

Cardiac hypertrophy is a critical component of phenotype in the failing heart. In recent years, accumulating evidence has demonstrated that oxidative stress plays important roles in the pathogenesis of myocardial hypertrophy either in response to chronic pressure overload or neurohumoral stimuli. In cultured myocardial cells, hypertrophy induced by angiotensin II, endothelin 1 or norepinephrine was found to be associated with increased ROS generation [10]. The potential mechanisms involved include ROS-induced activation of mitogen activated protein kinases and nuclear factor-κB. In pressure overload-mediated cardiac hypertrophy, NADPH oxidase existing in myocardial cells is the main source of ROS production and may result in various pathophysiological changes such as the activation of redox enzyme and progression to ventricular remodeling [11].

In the present study, oxidative stress was evaluated by detecting MDA, SOD, GSH-Px and ROS in the myocardium. Our findings revealed that ROS generation was significantly increased in the mouse model of cardiac hypertrophy. In addition, H_2_S was found to significantly attenuate oxidative stress in the myocardium of hypertrophic mice, which might be a critical protective mechanism against TAC-induced myocardial hypertrophy.

Nrf2 is an important regulator of endogenous antioxidant systems. Under normal conditions, Nrf2 locates in the cytoplasm and binds with Keap1 to mediate a rapid ubiquitination and degradation of Nrf2 by the proteasome. In response to oxidative stress, Nrf2 is released from Keap1 and transfers into the nucleus to correlate with ARE in the promoters of antioxidant enzyme regulatory genes [12]. In the present study, H_2_S was found to enhance the binding activity of Nrf2-ARE and increase the protein expression of antioxidant enzymes HO-1 and GCLM, which consequently augments the resistance to oxidative stress in the hypertrophic mice.

PI3K is a lipid kinase and generates PI(3,4,5)P3, which is a second messenger critical for the translocation of Akt to the cytoplasmic membrane. The activated Akt kinase plays crucial roles in various cellular functions such as cellular survival and proliferation by phosphorylating a number of substrates, including Ikappa B kinase, Bad, caspase-9 and forkhead transcription factors [13]. In the present study, our results suggested that PI3K/Akt signaling was suppressed in the myocardium of hypertrophic mice, while exogenous administration of NaHS was found to activate PI3K/Akt pathway.

To verify whether H_2_S regulates Nrf2 via PI3K/Akt-dependent pathway, we transfected cardiomyocytes with PI3K siRNA and then subjected them to Ang-II and NaHS. Our results revealed that suppression of PI3K/Akt signaling was associated with reduced nuclear accumulation of Nrf2 and elevated ROS production in cardiomyocytes treated with NaHS and Ang-II, which suggests that H_2_S could attenuate oxidative stress via PI3K/Akt-dependent activation of Nrf2 pathway.

In summary, our study reveals that H_2_S protects against TAC-induced cardiac hypertrophy by attenuating oxidative stress. The antioxidant roles of H_2_S in myocardial hypertrophy probably depend on the activation of PI3K/Akt pathway, which consequently increases Nrf2 activity and HO-1 and GCLM expression.

## MATERIALS AND METHODS

### Animal model

All experiments were carried out in accordance with the Guide for the Care and Use of Laboratory Animals. The TAC surgery was performed on 8 weeks old male C57BL/6 mice as previously described [14]. The sham group underwent a sham operation involving thoracotomy and aortic dissection without constriction of the aorta. The mice were divided into 4 groups: Sham group, TAC group, TAC+NaHS group (TAC mice were intraperitoneally administered with NaHS solution at a dose of 15μmol/kg/day) and NaHS group (sham mice were intraperitoneally administered with NaHS solution). After 4 weeks, animals were sacrificed by cervical dislocation, and the hearts were harvested for analysis.

### Cardiomyocyte culture

Neonatal ventricular myocytes were isolated from 1-2 days old mice. Briefly, myocardial tissue was surgically removed and then dispersed in a series of incubations at 37°C in D-Hanks buffered solution containing 1.2 mg/mL pancreatin and 0.14 mg/mL collagenase (GIBCO, USA). After centrifugation, the cells were suspended in Dulbecco's modified Eagle medium/F-12 (GIBCO, USA) containing 10% heat-inactivated foetal bovine serum, 100 U/ml penicillin, 100 μg/ml streptomycin, and 0.1 mM bromodeoxyuridine. The dissociated cells were preplated at 37°C for 1 h to separate cardiomyocytes by adherence of cardiac fibroblasts. The cardiomyocytes were then collected and diluted to 1×10^6^ cells/ml and plated in 1% gelatin-coated culture dishes.

### Measurement of H2S content

The H_2_S levels in myocardial tissue were measured by the methylene blue method which was described by Zhou et al. [15]. This method is based on the reaction of sulfide with N, N-dimethyl-p-phenylenediamine, in a ferric chloride catalyzed reaction with a 1:2 stoichiometric ratio to give the methylene blue dye, which is detected by spectrophotometry.

### Echocardiographic study

Cardiac hypertrophy was evaluated by echocardiographic analysis of heart size, including left ventricular posterior wall thickness at end-systole (LVPWs), left ventricular posterior wall thickness at end-diastole (LVPWd), interventricular septum thickness at end-diastole (IVSd) and interventricular septum thickness at end-systole (IVSs). All measurements were averaged for 3 consecutive cardiac cycles.

### Histological analysis

Left ventricular tissue was fixed in 10% buffered formalin, embedded in paraffin, and sliced into 5-μm-thick sections. Slides were stained with hematoxylin-eosin (HE) and observed under a light microscope. Myocyte cross-sectional area (CSA) was measured using Pro Plus 6.0 image analysis software.

### Measurement of oxidative stress

Oxidative stress was assessed by detecting malondialdehyde (MDA) levels, superoxide dismutase (SOD) and glutathione peroxidase (GSH-Px) activities and ROS generation in the myocardial tissue according to the instructions of detection kits (Jiancheng Biotech, China).

### siRNA transfection

Cardiomyocytes were seeded into 6-well plates for 24h and then transfected with NF-E2-related factor 2 (Nrf2) or PI3-kinase (PI3K) siRNA using Lipofectamine 2000 reagents. Subsequently, the cells were treated with angiotensin II (Ang-II) for 30 min after incubated with or without NaHS (100 μM).

### PI3K activity assay

PI3K activity was determined using an enzyme-linked immunosorbent assay (ELISA) kit (Echelon Biosciences, Salt Lake City, USA) following the manufacturer's instructions. In this method, PI3K activity was evaluated by detecting the conversion of PI(4,5)P2 into PI(3,4,5)P3.

### Real-time PCR

Total RNA was isolated from myocardial tissue using TRIzol reagent (Invitrogen, USA). RNA was reverse transcribed using SuperScript First-Strand cDNA System (Invitrogen, USA). Real-time reaction was run and analyzed using a real-Time PCR system (ABI 7300). The primer sequences used in this study were as follows: ANF, 5′-CTCCGATAGATCTGCCCTCTTGAA-3′ and 5′-GGTACCGGAAGCTGTTGCAGCCTA-3′; BNP, 5′-GCTCTTGAAGGACCAAGGCCTCAC-3′ and 5′-GATCCGATCCGGTCTATCTTGTGC-3′; β-MHC, 5′-CAGACATAGAGACCTACCTTC-3′ and 5′-CAGCATGTCTAGAAGCTCAGG-3′. The relative expression of mRNA was calculated using the comparative cycle threshold (CT) (2^−ΔΔCT^) method.

### Western blotting

The samples were homogenized in 0.1% SDS buffer and the lysates were centrifuged at 12,000 rpm for 15 min. The supernatant was collected and the protein concentration was determined. The extracted protein was separated on SDS-PAGE gel and transferred onto PVDF membrane (Millipore, USA). The membrane was blocked with 5% bovine serum albumin for 1h to reduce non-specific binding. Then, the blot was incubated with the primary antibody for 12h at 4°C. The antibodies used in this study were purchased from Cell Signaling Technology. After washing, the blot was incubated with HRP-conjugated secondary antibody (Santa Cruz, USA) for 1h at room temperature. Finally, the signal was detected using the enhanced chemiluminescence kit (Amersham Biosciences).

### Statistical analysis

In this study, statistical analysis was performed using SPSS software. The differences between two groups were determined by Student's t-test, and the differences among more than two groups were determined by Analysis of Variance followed by SNK-q method. *P*<0.05 was considered statistically significant.
